# Features of the triggering of the yellow code and factors associated with the occurrence of adverse events

**DOI:** 10.1590/0034-7167-2022-0181

**Published:** 2023-03-17

**Authors:** Pollyana Karine Lopes dos Santos, Gabriella Novelli Oliveira, Karina Sichieri, Diná de Almeida Lopes Monteiro da Cruz, Lilia de Souza Nogueira

**Affiliations:** I Universidade de São Paulo. São Paulo, São Paulo, Brazil; II Universidade de São Paulo, Hospital Universitário. São Paulo, São Paulo, Brazil

**Keywords:** Hospital Rapid Response Team, Emergency Treatment, Inpatient Care Units, Nursing Care, Vital Signs., Equipo Hospitalario de Respuesta Rápida, Tratamiento de Urgencia, Unidades de Internación, Atención de Enfermería, Signos Vitales., Equipe de Respostas Rápidas de Hospitais, Tratamento de Emergência, Unidades de Internação, Cuidados de Enfermagem, Sinais Vitais.

## Abstract

**Objective:**

to analyze the characteristics of the activation of the yellow code in wards and identify the factors associated with adverse events after the Rapid Response Team.

**Methods:**

a cross-sectional study with retrospective analysis of medical records of adults admitted to medical or surgical clinic wards of the University Hospital of São Paulo.

**Results:**

among the 91 patients, the most frequent signs of triggers (n=107) were peripheral oxygen saturation of less than 90% (40.2%) and hypotension (30.8%). Regarding the associated factors the research identified each minute of attendance of the Rapid Response Team in the wards increased by 1.2% odds of adverse events (twenty-four unplanned admission in the ICU and one cardiac arrest) in the sample (p=0.014).

**Conclusions:**

decreased oxygen saturation and hypotension were the main reasons for the triggering, and the length of care was associated with the frequency of adverse events.

## RESUMO

**Objetivo:**

analisar as características do acionamento do código amarelo em unidades de internação e identificar os fatores associados à ocorrência de eventos adversos após o atendimento do Time de Resposta Rápida.

**Métodos:**

estudo transversal com análise retrospectiva de prontuários de adultos internados em enfermarias de Clínica Médica ou Cirúrgica de hospital universitário de São Paulo.

**Resultados:**

entre os 91 pacientes, os sinais mais frequentes dos acionamentos (n=107) foram saturação periférica de oxigênio inferior a 90% (40,2%) e hipotensão arterial (30,8%). Quanto aos fatores associados, identificou-se que cada minuto de atendimento do Time de Resposta Rápida nas enfermarias aumentou em 1,2% a chance de ocorrência de eventos adversos (24 internações não planejadas em Unidade de Terapia Intensiva e uma parada cardiorrespiratória) na amostra (p=0,014).

**Conclusões:**

queda da saturação de oxigênio e hipotensão arterial foram os principais motivos de acionamento, e o tempo de atendimento foi associado à ocorrência de eventos adversos.

## RESUMEN

**Objetivo:**

analizar características de la activación del código amarillo en unidades de internación e identificar factores relacionados a ocurrencia de eventos adversos después de la atención del Equipo de Respuesta Rápida.

**Métodos:**

estudio transversal con análisis retrospectivo de prontuarios de adultos internados en enfermerías de Clínica Médica o Quirúrgica de hospital universitario de São Paulo.

**Resultados:**

entre 91 pacientes, los signos más frecuentes de las activaciones (n=107) fueron saturación periférica de oxígeno inferior a 90% (40,2%) y hipotensión arterial (30,8%). Cuanto a factores relacionados, identificado que cada minuto de atención del Equipo de Respuesta Rápida en enfermerías aumentó en 1,2% la chance de ocurrencia de eventos adversos (24 admisiones no planeadas en Unidad de Cuidado Intensivo y un paro cardíaco) en la amuestra (p=0,014).

**Conclusiones:**

caída de saturación de oxígeno e hipotensión arterial fueron los principales motivos de activación, y tiempo de ateción fue relacionado a ocurrencia de eventos adversos.

## INTRODUCTION

Cardiac arrest (CA), the unplanned admission to the Intensive Care Unit (ICU) and death are adverse events that can occur during the hospitalization of patients. Studies show that delays in the recognition of hemodynamic instability in patients may contribute to the occurrence of these events^(1-2)^ and, to avoid them, the surveillance of vital signs is fundamental since it makes it possible to identify those at risk of clinical deterioration^(1)^.

In this context, Rapid Response Teams (RRT) emerged to meet cases of clinical worsening and/or CA of hospitalized patients. Since then, the Agency for Healthcare Research and Quality (AHRQ) has guided two practices associated with patient safety: patients monitoring by early detection systems of severity; and the RRT use^(3)^. In clinical practice, the RRT can be composed of two types of teams: the specific one for patients in CA confirmed by the health team, called blue code; and the RRT that performs surveillance of patients with signs of clinical instability in wards^(1,4)^, known as yellow code. The latter aims to conduct clinical surveillance and early interventions for severe and unstable patients outside the ICU to avoid CA^(5)^.

A set of clinical signs and/or sudden changes in vital parameters may indicate the severity of the patient that needs immediate intervention. Therefore, the criterion for triggering RRT is usually based on changes in heart rate (HR), respiratory rate (RR), systolic blood pressure (SBP), and/or peripheral oxygen saturation (SpO2) of the patient. Other signs that indicate the activation of RRT are a sudden change in the level of consciousness, pallor, cyanosis of the extremities, acute pain of high intensity, convulsive crisis, and changes in capillary blood glucose^(4)^.

In this sense, the use of a strategy, such as RRT, awakens in the team the sense of surveillance of the clinical condition and vital parameters of patients hospitalized in wards. A Finnish study identified that, of the 1,914 patients evaluated, 564 had altered vital signs, with a higher number of RRT triggers in the presence of respiratory changes (n = 254; 45%); and, of the total of these patients, 112 (20%) were subsequently admitted to the ICU^(6)^.

In Brazil, The Heart Institute (InCor) of the Hospital das Clínicas of São Paulo was one of the first services to report the experience of RRT implementation. Five years after the start of RRT in the Institution, study results showed that nurses positively evaluated the program, and 42% of them reported that RRT should also be activated when the unit professional is concerned about any changes in the patient, regardless of stable vital signs^(7)^. Another investigation identified a similar finding in which 57 (38%) of the 150 yellow code triggers investigated were due to concern for the patient’s general condition, with no changes in vital signs^(8)^.

Researchers discuss the quality and frequency of vital signs records and their correct interpretation as factors that may influence the time to trigger RRT and, consequently, the patient’s clinical outcome^(9-10)^. However, waiting for the patient to present the criteria for triggering the RRT to take a course of action can lead to unfavorable outcomes^(10-11)^, which demonstrates the relevance of the clinical evaluation and perception of the nurse in the early recognition of the worsening of the patient’s clinical condition.

The literature offers little information about the characteristics of triggering the yellow code and variables that may influence the occurrence of adverse events after performing the RRT. In this sense, to identify findings that contribute to answers to knowledge gaps on the subject, this study is proposed guided by the following questions: What are the reasons for the yellow code triggers? What are the variables associated with adverse events that occurred immediately after RRT care that is component in the yellow code?

## OBJECTIVE

To analyze the characteristics of the activation of the yellow code for patients in inpatient units and identify the factors associated with the occurrence of adverse events after RRT care.

## METHODS

### Ethical aspects

The Research Ethics Committee of the institution approved the study, which, because it is a documentary research, was exempted from the application of the informed consent form to patients or family/legal guardian.

### Design, period, and place of the study

It is a cross-sectional study guided by the STROBE tool. It retrospectively analyzed medical records of patients admitted to a public, university hospital in São Paulo, Brazil, from April 30, 2018, to July 31, 2020. The institution has 28 inpatient beds in the medical clinic and 27 in the surgical clinic wards. The Yellow Code was implemented by the hospital’s Patient Safety Center in April 2018, initially in the surgical clinic ward, to identify surgical patients who presented clinical instability during hospitalization and needed early evaluation and treatment. In 2019, Yellow Code was expanded to the medical clinic ward.

The flowchart of the institution’s yellow code service defines the criteria for the nursing team to trigger the RRT for the care of patients admitted to the medical or surgical clinic wards. The presence of any sign of clinical deterioration of the patient in terms of RR, SBP, HR, SpO2, capillary glycaemia, and mental state, as well as the presence of chest pain, seizure, and/or significant bleeding, is an indicative criterion for immediate activation of RRT. In addition, the nurse’s perception of some aspects related to the clinical worsening of the patient may also justify the activation of the yellow code.

### Sample

The study sample consisted of medical records of patients older than or equal to 15 years admitted to the medical or surgical clinic wards in the period proposed in the research and who had the yellow code activated at least once during their stay in the unit.

### Study protocol

The dependent variable of the study was an adverse event (Death, unplanned admission to the ICU, or CA) that occurred immediately after the RRT care that composes the yellow code. The following independent variables were analyzed to identify the factors associated with the occurrence of these events: sex, age, age-adjusted Charlson comorbidity index (CCI) score^(12)^, time between admission to the unit and the activation of the yellow code (in days) and between the last measurement of vital signs and the activation of the code (in hours), signals of activation of the yellow code (RR < 8 breaths/minute or > 28 breaths/minute , SBP < 90 mmHg or >180 mmHg, HR < 40 bpm or > 130 bpm, SpO2 < 90%, acute change in mental status, seizure, acute significant bleeding, chest pain, capillary blood glucose < 60 mg/dL or > 400 mg/dL and/or others that encompassed conditions of clinical worsening recognized by the nurse), time between the activation of the yellow code and the arrival of the RRT (in minutes) and the attendance of the team in the unit (in minutes), number of conducts performed by the RRT, in addition to the National Early Warning Score (NEWS)^(13)^, referring to the vital signs measured before the activation of the code, that is, during the routine monitoring of the patient.

The NEWS is a tool to identify the clinical deterioration of the patient by analyzing the following physiological parameters: RR, SBP, HR, SpO2, whether the patient require oxygen therapy or not, in addition to the level of consciousness and body temperature. Its score ranges from 0 to 3 for each parameter evaluated, in which 0 is the value assigned to the signs within the normality, and 3, signs with serious alterations and requiring alertness. This scale guides the health team in the conduct and frequency of control of vital signs, being a helpful tool for surveillance of adult patients in wards and determination of the safe interval of control of vital signs. For this purpose, the suggested monitoring frequency according to the final NEWS score follows this order: 0 point - every 12 hours; from 1 to 4 points - between 4 and 6 hours; 3 in a single parameter or from 5 to 6 points - every hour; 7 points or more - continuous monitoring^(13)^.

Information on the reason for hospitalization according to the International Classification of Diseases (ICD-10) was retrieved from the patients’ medical records for sample characterization. In addition, the study collected data on the conducts performed by the RRT during the care, the type of exit from the unit and the condition of patients when leaving the hospital. Researchers created a data collection instrument, which directed the retrieval of information from the activation sheets of the yellow code of the units and the patients’ medical records to complement the research data, including the calculation of NEWS. The data was collected and stored in the Research Electronic Data Capture software (REDCap^®^), ensuring the security of the information collected^(14)^. The research created a pilot with data collection from ten patients who were subsequently included in the final sample of the study to verify the need for adjustments in the system. The tools used in REDCap^®^ were electronic collection, dynamic management, and export of data^(14)^.

### Analysis of results and statistics

Data was transferred from REDCap^®^ to a spreadsheet in the Microsoft Excel^®^ program and analyzed in the statistical program R (version 4.1.1) by a professional statistician. The study performed descriptive statistics with absolute and relative frequencies for categorical variables and means and standard deviation (SD) for numeric variables to characterize the sample. The research used the binary logistic regression to identify the factors associated with the occurrence of adverse events after RRT care. All independent variables of the study were simultaneously inserted in the model; and their predictive capacity, evaluated by the area under the curve Receiver Operator Characteristic (AUC-ROC). Variance Inflation Factor (VIF) was used to identify the presence of multicollinearity among the variables that remained in the final model, and VIF values lower than 5 indicated the absence of collinearity. The significance level adopted in all analyses was 5%.

## RESULTS

Among the 91 patients who made up the study sample, there was a predominance of hospitalization in the surgical clinic (97.8%) and ICU origin (30.8%), followed by the emergency room and surgical center (26.4% each). Table 1 shows the characteristics of the patients regarding sex, age, comorbidities, and reason for hospitalization.

**Table 1:** Patients (N = 91) according to demographic characteristics, comorbidities, and reason for hospitalization, São Paulo, São Paulo, Brazil, 2018-2020

Variables	n (%)	Average (SD^*^)
Sex		
Male	55 (60.4)	
Female	36 (39.6)	
Age		58.6 (19.5)
ICC score^†^		2.7 (2.5)
Reason for hospitalization		
Injury, poisoning and certain other consequences of external causes	34 (37.3)	
Diseases of the digestive system	30 (33.0)	
Neoplasms	8 (8.8)	
Diseases of musculoskeletal system	5 (5.5)	
Diseases of the respiratory system	5 (5.5)	
Diseases of the skin and subcutaneous tissue	3 (3.3)	
Diseases of the genitourinary system	2 (2.2)	
Others^‡^	4 (4.4)	

^*^ SD - Standard deviation; ^†^ CCI - Age-adjusted Charlson Comorbidity Index; ^‡^ Other: circulatory system diseases (n = 2), endocrine, nutritional and metabolic diseases (n = 1) and eye and related diseases (n = 1).

Of the total patients in the study, 83.5% had one yellow code trigger, and 16.5% had two or more in the same hospital stay, totaling 107 triggers in the sample. A decrease in peripheral oxygen saturation (40.2%) and hypotension (30.8%) were the most frequent signs of yellow code activation (Table 2). There was no case of triggering due to the presence of a convulsive crisis.

**Table 2:** Attendances (N = 107) according to yellow code trigger signals, São Paulo, São Paulo, Brazil, 2018-2020

Yellow Code trigger signals	n (%)^*^
Peripheral oxygen saturation < 90%	43 (40.2)
Systolic blood pressure < 90 mmHg	33 (30.8)
Chest pain	17 (15.9)
Acute change of mental state	17 (15.9)
Heart rate > 130 bpm	14 (13.1)
Respiratory rate > 28 breaths/minute	8 (7.5)
Systolic blood pressure > 180 mmHg	5 (4.7)
Significant acute bleeding	5 (4.7)
Heart rate < 40 bpm	4 (3.7)
Capillary glucose < 60 mg / dL	3 (2.8)
Respiratory rate < 8 breaths/minute	2 (1.9)
Capillary blood glucose > 400 mg / dL	1 (0.9)
Another trigger signal^†^	20 (18.7)

* *Allows more than one response;* ^**†**^ *Included dyspnea (n = 4), emesis (n = 2), low urinary output (n = 2), adventitious noises on pulmonary auscultation (n = 1), gastric reflux (n = 1), agitation (n = 1), anxiety (n = 1), distended abdomen (n = 1), occurrence with surgical wound (n = 1), respiratory failure (n = 1), occurrence with chest drain (n = 1), systolic pressure drop, but with a value greater than 90 mmHg (n = 1), pasty speech (n = 1), cold and sticky skin (n = 1) and intense sweating (n = 1).*

Times related to the activation of the yellow code and attendance of the RRT are described in Table 3.

**Table 3:** Times related to the activation of the yellow code and Rapid Response Teams care, São Paulo, São Paulo, Brazil, 2018-2020

Variables	Average	SD^^*^^	Median	Minimum	Maximum	CI95%^†^
Time between admission to the unit and the activation of the yellow code (in days)	5.2	7.5	2.1	0	38.8	4.0-6.9
Time between last vital sign measurement and yellow code activation (in hours)	4.9	3.6	4.0	0	15.0	4.2-5.6
Time between triggering the yellow code and the arrival of the RRT in the unit (in minutes)	12.9	24.3	10.0	0	175	9.5-19.7
Attendance time performed by RRT^‡^ in unit (in minutes)	58.3	66.2	30.0	0	280.0	47.3-72.6

^*^ SD - Standard deviation; ^†^ CI - Confidence interval; ^‡^ RRT - Rapid Response Team.

The average NEWS score calculated based on the vital signs that preceded the activation of the Yellow Code was 2.2 (SD 1.5). Table 4 shows that the most frequent procedures performed by RRT during care were oxygen therapy/airway approach (45.8%) and electrocardiogram (39.3%). Of the 35 cases involving conduct related to drug treatment, there was a higher frequency of analgesia and antibiotic therapy. The average number of procedures per attendance was 2.4.

**Table 4:** Attendances (N = 107) according to proceedings performed by the Rapid Response Teams, São Paulo, São Paulo, Brazil, 2018-2020

Proceedings conducted by RRT^^*^^	n (%)^†^
Oxygen therapy/airway approach	49 (45.8)
Electrocardiogram exam	42 (39.3)
Laboratory tests	35 (32.7)
Volume expansion	35 (32.7)
Drug treatment/blood transfusion	35 (32.7)
Imaging examinations	30 (28.0)
Change of decubitus	5 (4.7)
Fasting	4 (3.7)
Device insertion	4 (3.7)
Other proceedings^‡^	8 (7.5)

^*^ RRT - Fast Response Team; ^†^ Allows more than one response; ^‡^ It included: strict control of urinary output (n = 2) or of the drain (n = 1), enteroclism (n = 1), surgical revision (n = 1), compression of the drain orifice (n = 1), reinforcement of the dressing (n = 1) and placement of elastic stockings (n = 1).

Of the 25 attendances that had an adverse event as an outcome, 92.3% (n = 24) resulted in unplanned admission to the ICU and 7.7% (n = 1) in CA. There were no deaths at the end of the RRT care. The length of care provided by the RRT in the unit was the only factor associated with the occurrence of adverse events (unplanned admission to the ICU or CA) in the study patients. Each minute of RRT care increased the chance of unfavorable event occurrence by 1.2% (p = 0.014) in the sample. There was no multicollinearity among the variables that remained in the final model (VIF values lower than 5) (Table 5), and the model showed an excellent predictive capacity for the analyzed outcome (AUC-ROC: 0.830).

**Table 5:** Factors associated with the occurrence of adverse events in patients after Rapid Response Teams care, São Paulo, São Paulo, Brazil, 2018-2020

Variables	OR^^*^^	IC95%^†^	Valor de *p*	VIF^‡^
Male	0.654	0.177-2.386	0.516	1.444
Age	1.025	0.976-1.079	0.336	3.534
ICC score^§^	0.884	0.617-1.231	0.477	2.999
NEWS score^| |^	1.005	0.608-1.622	0.985	1.613
Time between admission to the unit and activation of the code (in days)	0.869	0.708-0.990	0.098	1.444
Time between last vital sign measurement and code triggering (in hours)	0.909	0.673-1.216	0.521	3.710
Trigger signal				
Respiratory rate < 8 breaths/minute	1.965	0.018-182.240	0.759	1.575
Respiratory rate > 28 breaths/minute	7.312	0.860-67.721	0.066	1.559
Systolic blood pressure < 90 mmHg	2.804	0.627-13.921	0.185	1.834
Systolic blood pressure > 180 mmHg	4.700	0.156-81.468	0.300	1.605
Heart rate < 40 bpm	8.084	0.439-147.890	0.141	1.593
Heart rate > 130 bpm	3.366	0.566-19.977	0.174	1.423
Peripheral oxygen saturation < 90%	1.679	0.468-6.044	0.421	1.408
Acute change of mental state	1.750	0.294-10.092	0.528	1.832
Significant acute bleeding	3.866	0.128-67.226	0.363	1.518
Chest pain	3.235	0.417-24.842	0.249	1.797
Capillary glucose < 60 mg / dL	3.205	0.079-93.780	0.495	1.820
Capillary blood glucose > 400 mg / dL	0.000	0.000-Inf	0.993	1.000
Another trigger signal	1.292	0.193-7.372	0.779	1.424
Time between code triggering and RRT^¶^ arrival in unit (in minutes)	1.000	0.975-1.023	0.981	1.315
RRT attendance time at the unit (in minutes)	1.012	1.003-1.023	0.014	1.610
Number of pipelines conducted by RRT^[Table-fn TFN6]^	1.208	0.735-1.991	0.452	1.496

* OR - Odds Ratio;

† CI - confidence interval;

‡ VIF - Variance Inflation Factor;

§ CCI - Charlson Comorbidity Index;

| | NEWS - National Early Warning Score;

¶ RRT - Rapid Response Team.

Regarding the type of exit from the ward, 50 (54.9%) patients were discharged from the hospital, 34 (37.4%) were transferred to the ICU at some point during their stay in the ward, 6 (6.6%) died, and 1 (1.1%) was transferred to another hospital. The hospital mortality rate was 16.5%.

## DISCUSSION

The yellow code and the performance of the RRT are relevant tools for the identification and immediate care of patients with signs of clinical deterioration in hospital wards that reduce cases of CA and in-hospital mortality^(1)^. A randomized multicenter study, a reference in the subject, showed that the most prominent role of the yellow code and the RRT is to identify early signs of clinical deterioration of patients and optimize proceedings before any potentially irreversible damage occurs. It observed that delays in this stage of clinical signs identification were associated with increased hospital mortality^(15)^. To date, the literature seeks to understand what factors affect the early identification of patients with signs of clinical instability that need to be attended by RRT^(1,15)^.

According to researchers, the highest code activation frequency is for patients admitted to the ward^(16)^, especially in the medical clinic, compared to the surgical^(1,16)^. In this study, there was a predominance of triggering for patients admitted to the surgical clinic ward. Characteristics of the patients and the hospital of the study may have influenced this result, such as the fact that the medical clinic has resident physicians 24 hours a day, which leads to a lower number of triggers in this unit.

A drop of SpO2, hypotension, chest pain, and altered level of consciousness were the most frequent reasons for the activation of the yellow code, with an average time of RRT of 58 minutes and a median of 30 minutes. A Portuguese study that characterized RRT care in a tertiary level University Hospital found similar results, in which the main reasons for triggering the code were altered breathing, observation by the nursing team that the patient was not well and altered level of consciousness, and the median time of care performed by RRT was 35 minutes^(17)^. The characteristics of the reasons for the triggers are based on the criteria of basic life support care (A = airway, B = breathing, C = circulation, and D = disability), and hospitals must make increasing efforts to train the health team in recognizing the signs of clinical deterioration and in early care^(17)^. Another highlight is the importance of nurses’ awareness to trigger when they notice that something is not right with the patient. A Brazilian study identified that the nurse’s concern with the general condition of the patient was the first reason for the activation of the RRT, surpassing the cases of alterations in vital signs^(8)^.

Research results showed that delays in care, i.e., time greater than one hour between the identification of signs of clinical deterioration and the activation of the yellow code, increased mortality in 30 days, ICU admission rate, and length of hospital stay of patients. The researchers stressed that the delays in triggering were due to the team’s perception that the change in question was not significant enough to be a reason for triggering^(18)^. It happens with the identification of signs of clinical deterioration secondary to hypoxia and hypotension. Even if they are reasons for triggering, often the ward team itself conducts procedures such as oxygen supplementation and volume replacement. In this way, the RRT is triggered only when the case worsens. In the previously mentioned study, the reasons for late triggers related to respiratory problems and hypotension were associated with increased hospital mortality^(18)^.

In the present investigation, the average time between the identification of signs of clinical deterioration and the arrival of RRT was 12.9 minutes, considerably longer than recommended by the institution (up to 5 minutes), and that found in the international literature was 2.5 to 4.5 minutes^(17)^. Another relevant point is the patients’ average NEWS score of 2.2, indicating that monitoring of vital signs should occur every four or six hours; and, in the sample, the time between the last measurement of vital signs and activation was, on average, 4.9 hours. It seems that the routine verification of vital signs of the inpatient units of the study was able to monitor the patient promptly, even without the daily application of a clinical deterioration detection system, such as NEWS, in the surveillance of signs of clinical deterioration.

The most frequent RRT proceedings and interventions cited in the literature are related to the priorities of care of the ABCD, such as oxygen therapy and volume expansion, corroborated by the conducts found in this research^(17-18)^. On average, there were 2.4 proceedings per activation of the RRT, and inferring that there was more than one priority of care while the ABCD was being performed. In addition, the fact that the length of RRT care was found to be a factor associated with the occurrence of adverse events (unplanned admission to the ICU and CA) demonstrates that, possibly in this sample, critically ill patients with yellow code activation required more than one RRT conduct and intensive care and, therefore, longer care time in the unit.

A recent systematic review of the literature aimed at verifying the outcomes of patients treated by RRT identified that more than half of the RRT calls resulted in hospitalization in the ICU since these patients had critical hemodynamic conditions and need for intensive support^(19)^. This review also showed that the mortality rate of patients treated by RRT is low and, generally, patients are discharged from the ward. In this sense, the researchers suggest that one of the most appropriate ways to verify mortality in studies on RRT is the measurement of mortality in 30 days and not the use of the hospital mortality rate^(19)^, reinforcing the need for other studies to analyze this outcome.

Finally, the use of the yellow code can improve the quality of hospital care and reduce the occurrence of aggravations and outcomes signs of clinical deterioration and immediate interventions to patients who present instability and greater severity, with particular attention to respiratory problems and hypotension.

### Limitations of the study

This study had performance in a single hospital and a small sample size as limitations, which should be considered in the generalization of the results. In addition, patients were monitored during the hospital stay, and the outcome of “death” was evaluated during this follow-up period. Therefore, it is suggested that other studies be conducted with longer follow-ups of patients treated by RRT, including the evaluation of mortality in 30 days.

### Contributions to the field of Nursing

The nursing team stays longer at the bedside and has a relevant role in identifying the signs of instability in patients^(20)^. The application of early detection of clinical deterioration tools such as NEWS has the potential to improve the early identification of signs of clinical deterioration and increase staff confidence in decision making^(21)^. In this way, the use of this tool can be a strategy to train the team regarding the warning signs of the yellow code^(18)^, decreasing the possible delays in its activation and, consequently, the occurrence of adverse events. In addition, nursing professionals need to develop observation skills, perception, and clinical judgment that something is not right with the patient, in addition to routine control and interpretation of vital signs because they are frequent findings of activation of the yellow code.

## CONCLUSION

The most frequent signs of the activation of the yellow code in the medical and surgical clinic wards were a drop in oxygen saturation and hypotension of the patients, being necessary interventions of the RRT in the approach of the airway, including oxygen therapy and electrocardiogram. The length of RRT care in the wards was the only factor associated with the occurrence of adverse events (unplanned admission to the ICU and CA) in the sample.

## SUPPLEMENTARY MATERIAL

https://doi.org/10.48331/scielodata.DLURTK
